# Radiation Therapy in Adult Soft Tissue Sarcoma—Current Knowledge and Future Directions: A Review and Expert Opinion

**DOI:** 10.3390/cancers12113242

**Published:** 2020-11-03

**Authors:** Falk Roeder

**Affiliations:** Department of Radiotherapy and Radiation Oncology, Paracelsus Medical University, Landeskrankenhaus, Salzburg 5020, Austria; f.roeder@salk.at; Tel.: +43-57-255-58923

**Keywords:** soft tissue sarcoma, radiation therapy, review

## Abstract

**Simple Summary:**

Radiation therapy (RT) is an integral part of the treatment of adult soft-tissue sarcomas (STS). Although mainly used as perioperative therapy to increase local control in resectable STS with high risk features, it also plays an increasing role in the treatment of non-resectable primary tumors, oligometastatic situations, or for palliation. This review summarizes the current evidence for RT in adult STS including typical indications, outcomes, side effects, dose and fractionation regimens, and target volume definitions based on tumor localization and risk factors. It covers the different overall treatment approaches including RT either as part of a multimodal treatment strategy or as a sole treatment and is accompanied by a summary on ongoing clinical research pointing at future directions of RT in STS.

**Abstract:**

Radiation therapy (RT) is an integral part of the treatment of adult soft-tissue sarcomas (STS). Although mainly used as perioperative therapy to increase local control in resectable STS with high risk features, it also plays an increasing role in the treatment of non-resectable primary tumors, oligometastatic situations, or for palliation. Modern radiation techniques, like intensity-modulated, image-guided, or stereotactic body RT, as well as special applications like intraoperative RT, brachytherapy, or particle therapy, have widened the therapeutic window allowing either dose escalation with improved efficacy or reduction of side effects with improved functional outcome. This review summarizes the current evidence for RT in adult STS including typical indications, outcomes, side effects, dose and fractionation regimens, and target volume definitions based on tumor localization and risk factors. It covers the different overall treatment approaches including RT either as part of a multimodal treatment strategy or as a sole treatment, namely its use as an adjunct to surgery in resectable STS (perioperative RT), as a primary treatment in non-resectable tumors (definitive RT), as a local treatment modality in oligometastatic disease or as palliative therapy. Due to the known differences in clinical course, general treatment options and, consequently, outcome depending on lesion localization, the main part of perioperative RT is divided into three sections according to body site (extremity/trunk wall, retroperitoneal, and head and neck STS) including the discussion of special applications of radiation techniques specifically amenable to this region. The review of the current evidence is accompanied by a summary on ongoing clinical research pointing at future directions of RT in STS.

## 1. Introduction

Radiotherapy (RT) has been an integral part of the treatment of adult soft-tissue sarcomas (STS) for many decades. While usually administered either pre-, intra-, or postoperatively (summarized as perioperative RT), its role as a definitive local therapy for primary tumors in medically or functionally inoperable patients as well as for the treatment of oligometastatic situations has recently gained attention due to clear improvements of the available radiation techniques. This seems also true for its palliative role given the growing possibilities of systemic approaches resulting in longer overall survival times in patients with metastatic disease.

This review aims at summarizing the current role of RT in adult-soft tissue sarcomas (STS). Bone sarcomas (including chondrosarcomas) and sarcomas typically arising in pediatric or adolescent patients (like Ewing sarcoma or rhabdomyosarcoma) as well as sarcoma-like lesions (for example desmoids, dermatofibrosarcoma protuberans, or chordomas) and sarcomas originating from visceral organs (like uterine sarcomas) are not covered. Because of the different behavior of adult STS in different body sites and the distinct differences in overall treatment approaches, the review is divided into seven sections, including perioperative RT in extremity/trunk STS, retroperitoneal STS, head and neck STS, definitive RT for primary tumors, SBRT for oligometastatic disease, palliative RT, and reirradiation of local recurrences. This will be accompanied by a section on future directions focusing on current clinical research directions.

## 2. Perioperative RT in Extremity/Trunk Sarcomas

**Figure 1:**
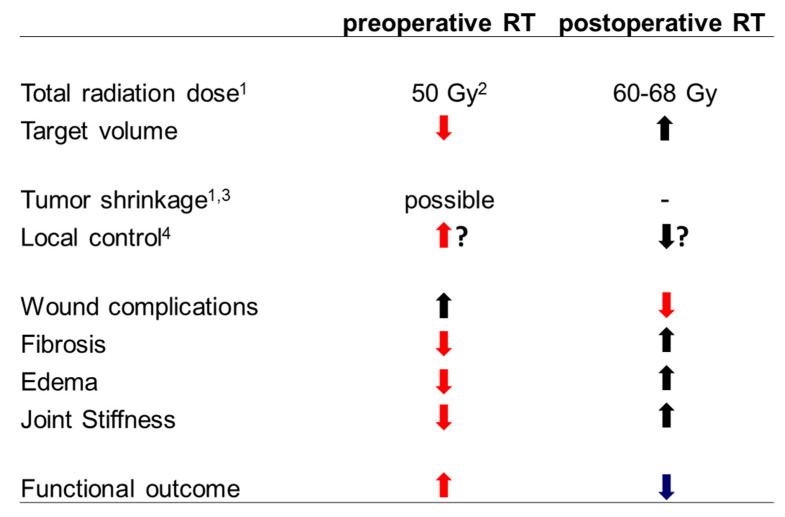
Pros and cons of preoperative and postoperative EBRT in extremity STS (modified from [6]). ^1^ For (anticipated) gross total resections without prior radiation therapy. ^2^ An additional preoperative or postoperative boost can be considered in selected cases. ^3^ Tumor shrinkage (downsizing) as volume reduction is possible according to the literature (see main text) if volume reductions transfer into improved resectability depends on the individual lesion. ^4^ Improved local control rates for preoperative compared to postoperative EBRT (but not vice versa) have been described in some series (see main text), although this finding is not supported by the only randomized trial.

The addition of RT to the surgical standard approach of wide resection undoubtedly results in increased local control based on randomized trials, meta-analyses, and large population-based studies in general [1,2,3,4], while its impact on survival is unclear [5,6]. However, the magnitude of the gain depends on several factors including resection margin, grading, size, localization, and histological subgroup. Thus, administration of RT must be weighed carefully against the anticipated acute and late toxicities with special regard to long-term functional outcome [4,7]. Data from the Scandinavian database indicates that the benefit in local control increases continuously with narrowing margins (especially if microscopic or even macroscopic residual disease is present or anticipated), higher grading and deep localization [4]. Small (<5 cm), superficial, low grade STS with wide margins represent the part of the spectrum with the lowest risk and the lowest benefit from the addition of radiation while large, deep seated, high grade STS with narrow or positive margins represent the part with the highest risk and benefit from adding RT [4]. Therefore, based on current international guidelines, the indication for additional RT is usually the strongest in patients with high grade lesions, while in low grade lesions RT is usually reserved for patients with (anticipated) positive margins or locally recurrent situations without prior RT [8,9]. Perioperative RT is usually administered via external beams (EBRT) and can be administered pre- or postoperatively. The only randomized trial directly comparing both approaches did not observe any significant differences in oncological outcomes but found distinct differences with regard to side effects [2,10]. While preoperative radiation therapy resulted in a doubled rate of major wound complications, all other side effects, especially late toxicities, were reduced compared to the postoperative approach. This is of major interest as late side effects like fibrosis, edema, and joint stiffness may severely affect the long-term functional outcome. The reduced late toxicity is probably related to the smaller irradiated volumes (due to easier target volume definition and the possibility for smaller safety margins) and the lower doses (probably due to a better tissue oxygenation) used during preoperative EBRT [10]. Preoperative radiation further includes the possibility of tumor shrinkage at least in radiosensitive histological subgroups [11] and prevents patients with postoperative complications from getting no radiation at all. A recent meta-analysis including eight trials with >1600 patients further described significantly increased local control and survival using the preoperative approach [3]. Therefore, current guidelines increasingly support the use of neoadjuvant EBRT [8], although the optimal timing should be evaluated individually. The pros and cons of preoperative and postoperative EBRT are summarized in Figure 1.

Technical optimization of EBRT with the introduction of intensity-modulated (IMRT) and image-guided radiotherapy (IGRT) has led to further improvements. Folkert et al. [12] observed a significant reduction of the local failure rate (8% vs. 15%) paralleled by a reduction of side effects in a large retrospective series comparing IMRT with 3D-conformal RT. In a follow-up study, they found a clear reduction in observed fractures after IMRT compared with the anticipated rate from a validated nomogram based on 3D-conformally treated patients [13]. O’Sullivan et al. [14] further showed in a phase II trial, that preoperative IMRT with sparing of anticipating skin regions of wound closure during surgery might result in less perioperative wound complications. Regarding target volume definition, international consensus guidelines have been published for pre- and postoperative EBRT [15]. Briefly, the gross tumor volume or the resection cavity is outlined on pre- or postoperative contrast-enhanced T1-weighted MRI. A safety margin to cover possible subclinical disease of 3–4 cm in longitudinal and 1.5 cm in axial direction is added (CTV). The CTV can be reduced at anatomical borders like uninvolved fascia or bone but should include any peritumoral edema based on T2-weighted MRI. An institutional margin is added to cover for set-up uncertainties (PTV). During RT planning, care should be taken to spare a longitudinal strip of skin and subcutaneous tissue from dose of more than 20 Gy to avoid lymph edema. The recommended dose for preoperative RT is 50 Gy. In the postoperative setting, 50 Gy are usually applied as a first phase of treatment followed by a cone down to a smaller volume that is boosted with 10–18 Gy according to margin status. Regarding treatment guidance, Dickie et al. [16] first demonstrated the possibility of using smaller safety margins resulting in smaller irradiated volumes by the introduction of daily IGRT. Recently, the results from RTOG 0630 indicated that even smaller target volumes might be possible without compromising local control if daily IGRT is used [17]. In this phase II trial, 86 patients with extremity STS received 50 Gy in 25 fractions to a reduced CTV covering the GTV with margins of only 3 cm longitudinally and 1.5 cm axially for high-grade or 2 cm longitudinally and 1 cm axially for low-grade tumors. The CTV–PTV margin was only 5 mm. Two-year local-recurrence free survival in patients undergoing surgery was 94%, but late grade2+ toxicities at 2 years including fibrosis, joint stiffness or edema were observed only in 11% of the patients (compared to 37% in the preoperative arm of the NCIC-trial using larger treatment volumes). However, a secondary analysis evaluating daily positioning errors clearly confirmed the need for daily image-guidance using such small margins [18].

Further fine tuning of radiation therapy with regard to an optimal functional outcome may be achieved by the use of alternative boosting techniques (IORT or brachytherapy) in patients at high risk for local failure [19]. Data from a recent European analysis demonstrated a 5-year local control rate of 86% combining EBRT with an IORT boost in an unfavourable patient cohort (29% R1 resections) with good functional outcome [20]. Aside from their high efficacy driven by the use of large single doses, IORT or brachytherapy boosts usually result in much smaller irradiated volumes compared to external-beam boosts, which seems to allow dose escalation without an increase in functional deficits by late side effects. Detailed descriptions of each technique including summarized results of the published series are beyond the scope of the current work but have recently been reviewed in [21,22]. Postoperative brachytherapy has been used also as a sole form of adjuvant radiation therapy based on the randomized trial by Pisters et al. [23] from the early 1990s, which showed improved local control rates compared to surgery alone. However, a large retrospective analysis comparing brachytherapy with IMRT found a significant local control advantage favoring IMRT, indicating that postoperative brachytherapy alone should be limited to selected cases [24].

## 3. Perioperative RT in Retroperitoneal Sarcomas

Retroperitoneal STS differ in many ways from extremity STS. They often present as large masses directly adjacent to vital structures in a region without clearly predefined anatomical compartments, which makes wide resection with large circumferential margins difficult [25]. Therefore, local recurrences and overall survival rates are clearly inferior compared to extremity STS. Based on these features, a clear rationale for the addition of radiation therapy exists. Taking the evidence from extremity sarcoma into account, in which the absolute benefit from radiation increases with smaller surgical margins, an even more pronounced advantage for additional radiation could be anticipated. Several retrospective series pointed at the direction of an increased local control rate by additional radiation, which was confirmed by a recent meta-analysis analyzing five studies with 803 patients (HR 0.47 favoring RT) [3]. A large, retrospective, multicentric French study including 537 patients and an international pooled analysis including 1007 patients both observed similar improvements in local control rates with the addition of radiation that were significant on multivariate analysis (HR 0.5 and 0.58) [26,27]. Moreover, two large population-based analyses including 2264 and 9068 patients even found a survival benefit for neoadjuvant radiation therapy compared to surgery alone in high grade STS [28,29]. However, population-based studies generally implicate a reasonable risk for selection bias and should be interpreted with caution.

Recently, the results of the randomized STRASS trial were published [30], which evaluated the value of neoadjuvant RT compared to surgery alone in 266 patients. As suggested from retrospective data, addition of neoadjuvant RT did not result in distinctly increased rates of severe side effects or major postoperative complications and did not significantly impact overall survival (3y-OS 85% vs. 84%) [30]. Surprisingly, the study also failed to show a benefit for neoadjuvant RT regarding the primary endpoint (3y-abdominal-recurrence free survival, ARFS) [30]. An event within ARFS was defined as either a true local recurrence after macroscopic complete resection, diagnosis of peritoneal sarcomatosis at laparotomy, macroscopic incomplete resection, local or distant progression according to RECIST criteria during radiation therapy, or a lesion becoming “inoperable” between randomization and surgery [30]. Subgrouping of the events in both arms revealed that the majority of events in the surgical only arm were true local recurrences after macroscopic complete resection (64%), while this was the case only for a minority of patients in the combined treatment arm (28%) [30]. The most common event within the combined arm was progression during radiotherapy according to RECIST criteria, although the value of RECIST criteria for response evaluation of STS after radiation or chemotherapy is highly controversial [31]. This prompted the investigators to perform two sensitivity analyses, excluding local progression during RT in the first, and local progression during RT or becoming medically unfit for surgery in the second, as an event in patients who achieved a macroscopic complete resection. This resulted in (non-significant) absolute 3-y-ARFS benefits of 7.3% in the first and 9.1% in the second analysis, favouring the combined treatment arm [30]. Further subgroup analyses revealed large differences based on histological subtype and grading. In the largest subgroup of liposarcomas (75% of all patients), neoadjuvant radiation resulted in a clear 3-y-ARFS benefit (64.7% vs. 60.4% in the overall analysis, 71.6% vs. 60.4% in the first sensitivity analysis and 75.7% vs. 65.2% in the second sensitivity analysis), while patients with leiomyosarcomas did not profit from radiation at all [30]. This finding might be explained by the different behavior of leiomyosarcomas inheriting a lower risk for local but a distinctly higher risk for distant failure compared to other STS subgroups, as shown by the same study group in a previously published large-pooled analysis [27]. Moreover, the STRASS trial observed a large benefit for additional radiation in low grade STS, while no distinct difference was found for intermediate grades and even a detrimental effect for high grade sarcomas seemed present [30]. However, low grade STS represented by far the largest group of the study population, while high grade sarcomas were clearly underrepresented, totaling only 12% (31 patients in both arms together) in the trial population, and systemic treatment was not part of the study. Interestingly, the group “not evaluable” for grading (which included more patients than the high-grade group) showed the largest benefit with preoperative RT, indicating that conclusions from these subgroup analyses should be drawn with caution. While the authors concluded that preoperative RT should not be part of the standard approach in treating retroperitoneal sarcomas, there seems to be a considerable role for RT at least in the subgroups showing favorable outcomes. Taking the other available evidence into account, further studies defining the role of RT especially in high grade retroperitoneal sarcomas in the context of perioperative systemic treatment seem warranted.

Aside from the discussion regarding the value of additional radiation per se, at least the question of optimal timing for perioperative radiation can be answered definitively in retroperitoneal STS in contrast to extremity STS. Due to the necessity to cover large volumes with directly adjacent radiosensitive organs at risk (like small bowel, liver, or kidneys), preoperative radiation clearly offers several advantages compared to a postoperative approach [32]:

- displacement of adjacent normal tissues by the tumor itself (tumor works as a spacer)
- lower total radiation dose (probably due to increased tissue oxygenation)
- more adequate target volume definition with more standardized, smaller margins
- reduced risk of tumor seeding during surgery
- fibrosis and thickening of a pseudocapsule
- tumor shrinkage (at least in radiosensitive subtypes)

Especially, displacement of adjacent organs at risk (which move to the resection cavity after primary resection und therefore would be irradiated to a large extent during postoperative radiation) and the lower total radiation dose needed lead to distinct reductions of dose to radiosensitive tissues with consequently reduced rates of severe side effects, just enabling radiation therapy with adequate coverage in many cases. Therefore, postoperative radiation is increasingly discouraged by major international guidelines [8,9].

Regarding radiation techniques, IMRT and IGRT should be preferred over conventional RT because sparing of normal tissue is even more crucial than in extremity STS. Dosimetric comparisons have shown clear advantages for IMRT with regard to small bowel and kidney doses [33] and a variety of clinical series have observed low rates of severe gastrointestinal toxicities (grade 3+ < 10%) using IMRT [32]. Considerable intrafractional (due to breathing) and interfractional variations (due to different organ fillings) can be observed, highlighting the need for motion management and daily image-guidance. For example, Wong et al. [34] calculated that a safety margin of 15 mm would be necessary to cover tumor movement due to different organ fillings without daily IGRT. Therefore, treatment planning should account for respiratory motion (for example by 4D-CT) and type of IGRT for accurate daily repositioning based on tumor and organ position (for example by daily cone-beam CT). Regarding target volume definition, international consensus guidelines addressing neoadjuvant RT have been published [35]. Briefly, the gross tumor volume (GTV) is outlined and expanded based on respiratory motion (ITV). An expansion of 1.5–2 cm in all directions is added to cover regions of subclinical disease (CTV). The margin can be reduced at anatomical borders like bone or encapsulated organs. Finally, an institutionally defined safety margin is added to account for daily positioning errors (PTV). The recommended dose for neoadjuvant treatment is 50 Gy, selective dose escalation to areas at high risk for margin-positive resections can be considered [36].

Similarly to extremity STS, further dose escalation can be achieved via alternative boosting techniques especially in patients at high risk for local failure. With IORT delivered via electrons or brachytherapy, surrounding radiosensitive organs at risk, such as the small bowel, can be surgically removed from the boost area, thus allowing dose escalation beyond the limits of EBRT [37]. Typical combination regimens use 45–50 Gy preoperative EBRT with 12–15 Gy IORT, which is equivalent to 70–80 Gy of conventionally fractionated RT. Several series including prospective trials reported encouraging results indicating increased local control with limited additional toxicity, but no randomized data is yet available [32,38,39,40,41,42]. Details regarding rationale, patient selection, techniques, and outcome are beyond the scope of this work but have been recently reviewed for example in [21]. Particle beams may also be used for further dose escalation because of their unique dosimetric advantages compared to photons. While no mature data from larger STS series on particle beams currently exists, preliminary results have already been published and several phase II trials are currently recruiting patients (see future directions section).

## 4. Perioperative RT in Head and Neck Sarcomas

STS are infrequently located in the head and neck area (5–15%) and most series report inferior overall survival rates compared to other body regions [43]. This might be due to the anatomic specificities of the head and neck region rarely allowing resections with wide margins given the proximity of vital structures. A different distribution of subtypes with known poor prognosis like angiosarcoma and MPNST seen more frequently in the head and neck region may also at least partly account for this finding [43]. Like in other locations, RT is frequently used as an adjunct to surgery to improve local control but is mainly applied postoperatively following the experience from other cancers of the head and neck region. The achievement of local control is of crucial interest in head and neck sarcomas for several reasons: a different pattern of relapse, and a lack of substantial salvage strategies. Similarly to retroperitoneal sarcomas, local recurrence rates are much higher than in extremity STS and usually exceed distant failure rates [44,45]. Major series describe locoregional relapse rates up to 54% [44] while distant failure after treatment for initially locoregional confined disease was reported in the range of only 9–31% [45]. Due to the limited salvage options, locoregional recurrence is directly related to overall survival [46] and represented the main cause of disease-related death in major series (65–74%) [47,48,49]. Therefore, the addition of RT to surgery seems highly justified based on the experience from other body regions [50]. However, given the rarity of the disease, no randomized data has been published evaluating the value of additional RT specifically in head and neck STS. Several retrospective series suggest a clear locoregional control benefit for the addition of radiation, even considering an imbalance of prognostic factors in favor of the surgery only groups. For example, Le et al. [51] reported 5-year local control rates of 59% for surgery alone and 77% for surgery and RT in a series of 65 patients, although the combined group included larger tumors and more incomplete resections. Eeles et al. [47] analyzed 130 patients, of whom 43 received surgery alone and 35 surgery and additional RT, and found that RT was a significant prognostic factor for local control (S:40% vs. S + RT:60%) according to multivariate analysis. Tran et al. [52] compared 94 patients treated with surgery with or without postoperative radiation and reported a significant difference in local control (52% vs. 90%) in favor of adjuvant radiation. They further analyzed their cohort according to margin status and found the most pronounced difference in patients with margin-positive resections. Recently, several large population-based analyses were performed to overcome the limitations of small patient numbers and to clarify the value of additional RT (although limited by the inclusion of varying proportions of pediatric sarcomas, bone sarcomas or sarcoma-like histologies). Cannon et al. [53] analyzed 1142 patients from the NCDB with margin-positive resections and found a significant benefit regarding 5-year-OS for the addition of radiation (57% vs. 48%). Subgroup analyses revealed that the benefit was found after microscopic and macroscopic incomplete resections with a similar magnitude (R1: 57% vs. 49%, R2: 57% vs. 41%) and was confirmed as an independent prognostic factor after controlling for other covariates which influenced OS according to multivariate analysis. Kim et al. [54] analyzed 1282 patients from the NCDB, of whom 401 received surgery and radiation therapy. Compared to the surgery only group, the use of RT was associated with poor grade and margin positivity. RT was applied preoperatively in 6% and postoperatively in 94% with no difference in overall survival according to timing of RT. Finally, Mahmoud et al. [55] analyzed 2493 patients with surgically treated, non-metastatic sarcoma of the head and neck region. Additional RT was applied in 41%, associated with larger tumor size, high grade, and positive margins. In the subset of high-grade patients (*n* = 788), utilization of RT increased to 53%. Again, patients with large tumor size or margin-positivity were more likely to receive RT, which was delivered preoperatively in 7% (median dose 50 Gy) and postoperatively in 93% (median dose 60 Gy). Perioperative RT significantly increased 5-year OS from 44% to 49% in the high-grade subgroup, which was confirmed in propensity score model based matched-pair analysis adjusting for covariates. In summary, perioperative radiation should be generously considered in high grade STS and regardless of grade in patients with (anticipated) margin-positive resection. Timing of perioperative EBRT does not seem to influence oncological outcome based on the available data; however, most patients have been historically treated with postoperative EBRT. Regarding target volume definition, no international consensus exists. In most series, the primary tumor region or the postoperative tumor bed has been treated with margin of 1.5–2 cm to form the CTV plus an institutionally defined PTV margin. Given the low rates of lymph nodes involvement (7–10%) [52,56,57], elective nodal irradiation does not seem necessary, similarly to the treatment of STS arising from other body regions. Typical doses have been adopted from the extremity sarcoma experience; therefore, around 50 Gy have been usually applied for preoperative and 60–66 Gy for postoperative EBRT. The use of modern radiation techniques like IMRT and IGRT seems advisable given the overwhelming body of evidence suggesting clear benefits in terms of acute and late toxicities compared to conventional techniques in other head and neck cancers treated with similar doses [58], although no data addressing this issue specifically for head and neck STS has been published so far.

## 5. Definitive RT for Inoperable Primary Tumors

Surgical resection with or without perioperative radiation is clearly the treatment of choice in the vast majority of patients with locoregionally confined disease and inherits the highest possibility for cure. However, in certain situations, surgery may not be feasible, for example in technically not resectable lesions (located usually outside the extremities) or in patients medically unfit for major surgery. In those situations, definitive radiation treatment should be considered as an alternative local treatment although it may not replace surgery in patients amenable to complete resection, even if this requires complex and/or functionally suboptimal resections like amputations. Early experience with definitive RT were presented in the late 1980s. Tepper et al. [59] reported on 51 technically or medically unresectable patients without distant metastases treated with conventional fractionated RT up to 64–66 Gy. Overall, 5-year-LC and -OS rates were 33% and 25%. Doses ≥ 64 Gy (5y-LC 44%) and smaller tumor size (crude LC < 5 cm: 88%, 5–10 cm: 53%, >10 cm 30) were associated with improved LC rates. Slater et al. [60] reported on 57 patients with gross residual disease after surgery or biopsy only proven unresectable STS treated with photons (79%) or combinations of photons and neutrons (21%). With a median dose of 61 Gy, they observed an overall 5-year-LC rate of 29%. Doses ≥ 65 Gy and small tumor size again were associated with improved LC. Major complications occurred in 8% mainly as soft-tissue necrosis or bowel obstruction. The complications rate was clearly dose dependent (<70 Gy: 2% vs. ≥70 Gy: 28%) while no difference for outcome or toxicity was found between photons only and combination with neutrons. However, those early experiences have a limited value for actual decisions because of the outdated radiation techniques and the inclusion of rhabdomyosarcomas, desmoids or other Non-STS histologies. The largest series, which included patients treated with more modern techniques like 3D-conformal RT, was published by Kepka et al. in 2005 [61]. They treated 112 patients with primary or recurrent unresectable STS excluding pediatric or Non-STS histologies. The median total dose was 64 Gy, mainly applied in conventional fractionation. With a median f/u of 29 months (139 mo in survivors), they observed 5-year rates LC, DFS and OS rates of 45%, 24%, and 35%. All endpoints were significantly affected by tumor size and total dose. While patients with tumors < 5 cm or 5–10 cm showed 5-y-LC rates of 51% and 45%, the rate dropped to 9% in patients with tumors > 10 cm. 5-year LC, -DFS and OS were significantly increased in patients receiving ≥ 63 Gy (LC: 60% vs. 22%, DFS 36% vs. 10%, OS 52% vs. 14%). Major complications were observed in 14% and were clearly associated with radiation dose. Patients who received total doses ≥ 68 Gy had a 22% complications rate compared to 8% in patients with less dose, which also occurred earlier. Based on this data, definitive RT seems able to achieve long-term local control in a substantial proportion of patients, especially in smaller lesions. Dose escalation above 63–65 Gy improved local control while doses ≥ 68–70 Gy resulted in clearly increased complication rates [59,60,61]. Modern radiation techniques like IMRT with IGRT or particle therapy offer clearly superior dose distributions, which (at least theoretically) will allow further dose escalation with acceptable toxicity; however, only a few reports on adult STS treated with those techniques have been published so far. Kamada et al. [62] reported early promising experiences with carbon ions. They treated 57 patients (64 lesions) with inoperable bone or soft tissue sarcomas in a phase I/II dose escalation trial. Total dose was stepwise increased from 52.8 to 73.6 GyE in 16 fractions. Local control was achieved in 12 of the 18 soft tissue sarcomas lesions. Acute and late grade 3+ toxicity was observed in 13% and 10%, mainly attributed to the highest dose level. Serizawa et al. [63] reported their experience with carbon ions for retroperitoneal sarcomas. They treated 24 inoperable patients with definitive RT using doses between 52.7 and 73.6 GyE in 16 fractions. Although one third of the patients had already recurrent tumors, they found a remarkable 5-year LC- and OS rates of 69% and 50% with no grade 3+ toxicities with a median follow-up of 36 months. Recently, Imai et al. [64] reported the largest series focusing on carbon ion treatment for unresectable localized axial STS. They treated 128 patients with a median dose of 70.4 GyE in 16 fractions and observed a 5-year-LC and OS rates of 65% and 46%. Late grade 3+ side effects were found only in 3%. Interestingly, no factor influenced local control significantly according to multivariate analysis; however, clear absolute differences were found according to histology. While liposarcomas did best (5-year LC 90%), MPNST showed the worst local control (5-year-LC 42%) with undifferentiated (UPS) and synovial sarcomas in between (5-year LC 66% and 52%).

Another possibility is to combine definitive RT with radiosensitizing agents. Historical data evaluating the combination of definitive RT with misonidazol, bromodeoxyuridine, or iododeoxyuridine reported crude local control rates of 60–86% [65,66], but were not further evaluated. Combination of RT with razoxane was evaluated in a randomized phase II trial including 82 patients with gross disease and resulted in significantly improved response rates (74% vs. 49%) and crude local control rates (64% vs. 30%) compared to radiation alone [67]. More recently, chemotherapy agents with known activity in metastatic STS like doxorubicine, ifosfamide, or temozolomide gained new attraction within combination regimens based on experiences in the neoadjuvant setting, which at least allowed assumptions of safety and efficacy also for definitive treatments. Pisters et al. [68] treated 27 resectable patients in a phase I/II trial with preoperative RT to 50 Gy and concurrent doxorubicin. Although started with a typical phase I design with planned escalated doses (4 mg/m^2^ bolus at day 1, followed by 12.5, 15, 17.5, or 20 mg/m^2^ continuous infusion over 4 days per week), the majority of patients (*n* = 22) were treated at the 17.5 mg/m^2^ level. Dose limiting toxicity was observed in 30% of the patients (grade 3 dermatitis). All but one patient subsequently underwent surgery with two showing a complete pCR and 11 patients showed more than 50% tumor necrosis. Cormier et al. [69] reported 43 patients treated preoperatively or definitively with a median dose of 50.4 Gy and concurrent ifosfamide. Median ifosfamide dose was 10 g/m^2^ per cyle with 40% receiving only one but 60% two or more cycles. Acute toxicity was scored as grade 3 in 29% and grade 4 in 22%. Of the resected patients, 14% showed pCR and 43% near pCR, indicating high efficacy at the cost of considerable toxicity. Unfortunately, no response assessments or local control rates were reported for the definitively treated patients. Sauer et al. [70] treated 23 patients with borderline resectable STS with neoadjuvant RT (50.4–69 Gy) and concurrent doxorubicin (50 mg/m^2^ d2 and d30) and ifosfamide (1.5 g/m^2^ d1–5 and d29–33). Acute toxicities were high, including 48% grade 3+ leucopenia and 26% grade 3+ dermatitis. One patient died due to pneumonia. Of 21 ultimately resected patients, 19% showed T downstaging and 14% pCR, which does not seem to be superior to the data observed with ifosfamide alone.

Data specifically focusing on patients treated definitively are even rarer. For example, Eckert et al. [71] reported a small series of 11 patients suffering from high-grade unresectable STS of head and neck or trunk treated with definitive RT and concurrent ifosfamide. Two patients additionally received hyperthermia. Ifosfamide was given at 1–1.5 g/m^2^ in 5 consecutive days in the first and fifth week of RT. Median RT dose was only 60 Gy but actuarial 5-year LC was 70% with a median f/u of 55 months. Only 18% of the patients developed local relapse while 5-year OS (34%) was limited mainly due to development of distant failure (54%). Acute toxicity was increased compared to historical data for sole RT (grade 3+ 55%) but seemed manageable. Jakob et al. [72] reported a series of 15 patients with locally-advanced, non-metastasized STS judged either unresectable or only at the cost of major functional deficits. They were treated with concurrent chemoradiation including IMRT with 50.4 Gy and temozolomide. Manageable grade 3 acute toxicities were observed in 27% with no grade 4/5 toxicities reported. Gross total resection was finally achieved in nine patients (60%), with seven of them being microscopically complete.

In summary, definitive RT offers the possibility for long-term local control and overall survival in a substantial proportion of patients with unresectable primaries. As dose escalation using modern radiation techniques including particle therapy and/or combination with systemic agents may widen the therapeutic window and may achieve an improved outcome, treatment decisions should be made by a multidisciplinary team on an individual basis taking tumor size and tolerance of adjacent organs at risk into account.

## 6. Definitive RT in Oligometastatic Situations

About 25–40% of patients with STS will develop distant metastasis even in case of optimal treatment regarding the primary [73]. The most common site for distant metastasis in the lungs (70–80%) followed by bone, liver, and brain [73]. Although the primary treatment in metastatic situations is systemic therapy, increasing evidence suggests a benefit of locally ablative treatments at least in the oligometastatic state, which prompted a general shift in treatment paradigms towards aggressive local therapies. Recently, a large observational study (METASARC) including 2165 patients with metastatic STS showed a clearly prolonged time to next treatment and improved overall survival in patients who received locoregional treatment (surgery, RT, or RFA) directed to their metastases according to multivariate analysis [74]. Falk et al. [75] further analyzed 281 STS patients with oligometastatic disease, of whom 164 received local ablative treatments including RT with a minimum EQD2 of 50 Gy. They observed a clearly improved median OS of 45 months with local treatment compared to 13 months without. Moreover, they proved a benefit from local treatments regardless of an association with chemotherapy. One possibility for locally ablative treatment aside from surgery or radiofrequency/microwave ablation is stereotactic body radiation therapy (SBRT). This radiation technique delivers large doses of highly focused radiation to small volumes in a few fractions (usually 1–8), resulting in much higher biologically effective (so-called ablative) doses (BED) than conventionally fractioned RT. It is generally amenable to metastases in different body sites including lung, liver, bone, brain, lymph node, or soft-tissue, and limited only by directly adjacent organs at risk of low radiation tolerance (for example spinal cord) or the number of metastases. A recent randomized phase II trial (SABR-COMET) compared oligometastatic patients (defined as 1–5 metastases with a maximum of 3 in one organ) of different histologies treated with standard palliative care with or without SBRT of all lesions, and observed a significantly improved 5-year OS favoring the SBRT arm [76,77].

### 6.1. Lung Metastases

The general efficacy of SBRT for lung lesions has been proven, for example in early stage lung cancer and lung metastases of different origins, resulting in high local control rates that were similar if not superior to surgical approaches [78,79,80]. Given the rarity of STS per se, data on its specific use in metastases from STS is limited. Major published series [81,82,83,84,85,86,87,88], (Table 1) included 15–52 patients with 25–117 lesions of mixed histologies (bone and soft-tissue sarcomas) usually not amenable to surgery but heavily pretreated in many cases. Dose and fractionation regimes slightly varied with total doses of 30–60 Gy in 1–10 fractions dependent on size and localization, but mainly achieved ablative BEDs of >100 Gy. Reported 1- and 2-year local control rates were 94–100% and 86–100% with barely grade 3+ toxicities. Reported 2-year OS rates (43–96%) strongly depended on selection but seemed comparable to well selected surgical series (3-year OS 21–71%) [84]. Moreover, long-term survival was observed in a substantial proportion of patients (up to 61%). A recent systematic review compared the efficacy of metastasectomy (MTS) with SBRT including 15 MTS and 6 SBRT studies totaling for 1104 MTS and 202 SBRT patients [89]. They observed a similar cumulative median OS (47 vs. 48 months) with only three patients (1.5%) suffering from grade 3+ toxicities in the SBRT group [89]. In summary, SBRT seems to be an equally effective, highly tolerable, alternative local treatment of a limited number of lung metastases (in most studies the number was restricted to 3–4) in patients with soft-tissue sarcoma especially if surgery is not an option due to functional or medical inoperability.

### 6.2. Bone Metastases

Bone metastases represent the second most common site of distant failure in STS patients [73]. They may cause severe restrictions to the quality of life due to pain or local complications like fractures and/or neurological complications, especially if located in the spine. Therefore, they were usually targeted by RT with palliative doses, which resulted in short-term pain control and in some cases durable recalcification, but its effects often lasted only for some months. As SBRT offers the precise application of higher effective doses aiming at durable control, increasing interest has been paid to this technique, especially because complete resection is not an option in the majority of cases. Folkert et al. [90] reviewed the MSKCC experience in 88 patients with 120 spine lesions treated either by single fraction stereotactic radiosurgery (SRS) with a median dose of 24 Gy or hypofractionated SBRT with median 28.5 Gy in 3–6 fractions. With a median f/u of 12 months, they observed 1-year local control and overall survival rates of 88% and 61%, which is superior to the published results with palliative treatment. Only 1% and 4.5% of the patients suffered from acute or late grade 3 toxicities with no grade 4/5 toxicities. Bishop et al. [91] reported on 48 patients with 66 spine lesions treated with different fractionation schedules. 1-year local control and survival for the entire cohort were 81% and 67% with no acute grade3+. Subgroup analysis revealed a favourable outcome in single vs. multiple vertebral body affections (1-y-LC 93% vs. 50%) and a more durable control with BED > 48 Gy (3-y-LC 66% vs. 20%). Late toxicities included 6 insufficiency fractures but no grade 3+ neurological toxicity. In summary, SBRT represents a valuable option for local ablative treatment of bone (especially spine) metastases in STS patients with a limited number of lesions. Care should be taken with regard to epidural involvement which usually requires schedules using more fractions and smaller single doses.

### 6.3. Other Sites

Generally, SRS or SBRT can be used also for metastases in different body regions, for example liver, brain, lymph nodes, or soft-tissue metastases. Several prospective trials and large retrospective series have established its role especially in brain metastases, liver metastases and (to a lesser extent) for lymph node metastases [92,93,94], consistently showing high local control rates with low toxicity. Due to the relative rarity of STS per se, most of these reports include only a minority of STS cases and very rare data on SBRT specifically addressing STS metastases has been published so far. For example, Sim et al. [95] analyzed 24 patients with 58 brain lesions of STS treated with stereotactic radiosurgery (median 19 Gy in one fraction) or fractionated stereotactic RT (25–35 Gy in 3–5 fractions). They observed one-year LC and OS rates of 89% and 38%. Yaeh et al. [96] further compared SBRT treatment of brain metastases from “radiosensitive” and “radioresistant” histologies (the latter including STS) and found no difference in local control (90% both). Therefore, SBRT can be considered in selected patients not amenable to surgery as an individual approach based on the promising results of the overall series.

## 7. Palliative RT

Standard treatment for patients with locally advanced (unresectable) or metastatic STS is typically systemic therapy based on histology. However, many patients develop severe symptoms like pain or local complications due to uncontrolled progressions of localized lesions, which are generally amenable to palliative RT. According to a recent survey from Australia, 37% of locally advanced or metastatic STS patients received palliative RT during their course of disease [97], mainly to symptomatic lesions of bone and lung or mediastinum. Although this comprises a substantial number of patients, the literature regarding efficacy, side effects, and optimal dose and fractionation schedules specifically for STS patients is very scarce. Instead, dose and fractionation follow the general rules for palliative RT established for different body sites based on major series, which included a variety of tumor entities, rather than focusing on a specific type of cancer. This leads to a variety of used fractionation concepts which only have in common, that typically larger single doses (hypofractionated RT) in a small number of fractions are applied to comparably low total doses. Tween et al. [98] reported the far largest series specifically addressing palliative RT in sarcoma patients. They included 105 patients with 137 treated lesions (114 STS, 23 bone sarcomas), which have been located in 15 different body sites and were treated with 25 different fractionation regimens including total doses of 8–60 Gy in 1–28 fractions. The largest subgroup were bony spine lesions (23%), the most common symptom was pain (70%) and the most common fractionation was 20 Gy in 5 fractions (26%). In the overall cohort, they observed symptomatic improvements at three months from RT in 70% of all STS and 55% of all bone sarcoma patients. In an effort to compare the efficacy of different fractionation regimes, they calculated the BED based on an alpha/beta value of 4. They observed an increase in symptomatic improvement rate for lower doses, which plateaued at a BED of 50 Gy. Indeed, the symptomatic improvement rate for patients who received 5 × 4 Gy (BED 40 Gy) was only 50% for STS patients compared to 70% in the entire cohort. Soyfer et al. [99] presented their experience using hypofractionated RT with even higher doses. They treated 17 patients with 39 Gy in 13 fractions (BED 68 Gy with alpha/beta 4) and reported durable pain control in 80% of the patients after 6 months. Moreover, none of the patients developed toxicities > grade 1. Jansen et al. [100] addressed the question of palliative RT in metastatic spinal cord compression from STS metastases in 4 patients treated with 5 × 4 or 10 × 3 Gy. They observed complete or partial pain relief at one month in 75% but no improvement in motor function at all. Merimsky et al. [101] evaluated the same issue in 19 patients treated with 10 × 3 Gy. Similarly, they observed allevation of pain, sensory deficits, motor deficits and sphincter deficits in decreasing percentages (87%, 53%, 43% and 0%). In summary, palliative radiation therapy may achieve short-term symptom control (especially pain control) in the vast majority of patients with symptomatic lesions and should therefore be considered widely in such cases. Based on the mentioned data, moderate dose escalation using slightly longer schedules may result in a higher symptom improvement rate and a more durable symptom control and should be considered in selected cases.

## 8. Reirradiation of Non-Metastatic, Locally Recurrent Primaries

Local recurrences of STS represent a generally challenging situation. Recurrences tend to have per se worse local control [102], indicating the general need for intensified therapy, but the initial treatment course usually has already exploited much of the therapeutic window. Therefore, treatment options depend strongly on tumor localization and prior treatment and should generally be assessed by a specialized multidisciplinary team for the individual patient. In previously non-irradiated recurrences, treatment should follow the principles of untreated primaries, although additional radiation might be considered more generously [8].

In previously irradiated recurrences, reirradiation can be considered especially in patients with longer time intervals between initial treatment and recurrence after meticulous assessment of the prior RT treatment plans with regard to doses in organs at risk. Care should be taken to limit the reirradiated volume and the cumulative doses to organs at risk to the possible minimum to avoid major late complications. This includes the use of modern EBRT techniques like IMRT with IGRT, particles and alternative RT techniques like brachytherapy alone or in combination with EBRT.

Due to the rarity of STS per se, literature regarding reirradiation is comprehensibly scarce and published results are difficult to interpret because of the inclusion of different locations and treatment combinations in comparably small cohorts. Comparative series mainly show increased local control by the addition of reirradiation to conservative surgery at the cost of increased acute and late toxicity. For example, Catton et al. [103] compared 11 patients treated by wide excision alone to 10 patients treated with wide excision followed by reirradiation delivered by EBRT, brachytherapy or both to a median cumulative dose of 100 Gy. They observed a significantly improved local control rate of 100% with reirradiation compared to only 36% with surgery alone after a median follow-up of 24 months. Although 60% of the reirradiated patients showed severe late complications, functional outcome was scored as good in 70% of them. Moureau-Zabotto et al. [104] reported on 83 patients with first local recurrences, of whom 38 patients received surgery alone, 25 surgery and additional RT without prior RT, and 20 surgery with reirradiation mainly via brachytherapy to cumulative doses of 95–115 Gy. Local control after a median follow up of 59 months was significantly improved by additional RT (64% vs. 45%) with an overall 5-year OS of 54%. In previously irradiated patients, the local control benefit from reirradiation with brachytherapy appeared to be even higher (63% vs. 26%). Late toxicity grade 3+ was scored in 49% of the patients with all grade 4 late complications (9%) attributable to reirradiation. Torres et al. [105] analyzed 62 patients with isolated first local recurrences of STS after conservative surgery and EBRT treated by wide excision with or without additional RT delivered mainly via brachytherapy implants. In contrast to others, they did not observe a significant improvement of local control by additional RT (5-year LC 58% vs. 39%) but reported significantly increased late complications (76% vs. 16%).

Regarding the optimal RT technique for reirradiation, brachytherapy might offer valuable benefits compared to EBRT regarding the limited irradiated volume [106]. Several series reported high local control rates with acceptable late toxicities. Pearlstone et al. [107] analyzed 26 previously irradiated patients (median dose 56 Gy) treated with brachytherapy to a median dose of 47 Gy and observed a 5-year local recurrence free survival of 52% with only 19% severe late complications. Nori et al. [108] reported 40 previously irradiated patients who received brachytherapy to a median dose of 45 Gy with a 5-year LC rate of 68% and late complications requiring surgery in 13%. Fontanesi et al. [109] treated 31 patients with either EBRT, brachytherapy, or the combination of both, and reported less late toxicity for patients receiving brachytherapy alone. In contrast, Indelicato et al. [110] found a 50% serious complication rate for patients reirradiated by EBRT with or without a brachytherapy boost. They further observed a higher risk for amputation due to complications if reirradiation doses exceeded 60 Gy or cumulative doses exceeded 111 Gy. If EBRT is considered, preoperative treatment might be preferable to postoperative RT given the usually smaller irradiated volumes and the lower doses needed for equal efficacy based on the experience from primary disease. Essner et al. [111] compared preoperative hypofractionated RT combined with intraarterial chemotherapy to postoperative conventionally fractionated RT in a small series of previously irradiated recurrent extremity STS. They found improved local control rates (86% vs. 29%) and less major late complications (43% vs. 50%) with consecutively higher limb-preservation rates (86% vs. 36%) favoring the preoperative approach. Finally, particle therapy should be considered because of its general ability to limit dose to surrounding tissues. Guttman et al. [112] reported a prospective series of 23 patients (mainly non-extremity lesions) treated with perioperative or definitive re-irradiation with protons (50–74 Gy) and observed a 3-year local failure rate of 41% with only 15% grade 3+ late toxicities.

In summary, reirradiation in locally-recurrent patients probably results in increased local control compared to conservative surgery alone but has to be weighed against treatment-related late complications for the individual patient. RT techniques with limited irradiation volumes like brachytherapy or particle therapy might be preferable options with regard to functional outcome.

## 9. Future Directions

Current research on radiation therapy in STS is aiming in different directions. In extremity sarcomas, local recurrences rates are already comparably low with the current treatment approaches. Therefore, further treatment optimization aims at reducing treatment-associated acute or late sequelae. Based on the findings from photon RT treatments demonstrating that smaller irradiated volumes lead to reduced late side effects and on the promising preliminary experiences from other body regions, several institutions are currently evaluating particle beams for perioperative RT in extremity STS. For example, investigators from Loma Linda launched a phase II trial investigating preoperative proton therapy with 50 GyE followed by limb-sparing surgery, with the rate of grade 2+ late toxicity at 2 years as the primary endpoint (NCT NCT01819831). Another area of investigation for treatment optimization involves shortening radiation schedules in order to save resources and to improve access for patients living in areas with limited radiation therapy facilities. The encouraging results from other tumor sites such as rectal cancer [113] and the evidence for alpha/beta values below 10 Gy in STS [114,115] has increased interest in hypofractionated RT schedules for extremity. First results have been published by the Polish group, investigating preoperative EBRT in 272 unselected patients using a 5 × 5 Gy schedule given in one week followed by immediate surgery in resectable extremity and trunk STS [116]. In primary cases, they observed three-year LRFS and OS rates of 81% and 69%, which seem slightly inferior compared to the series using conventional fractionation [12]. However, they also reported low rates of acute and late toxicities; only 7% needed secondary surgery due to wound complications compared to 16% in the preoperative arm of the NCIC trial. Similar results have been observed for grade 2+ late edema and fractures, indicating that short-course irradiation might be suitable at least for subgroups of patients. Kubicek et al. [117] recently reported the results of a phase II trial including 13 patients treated with preoperative SBRT. Most patients received 35 Gy in 5 fractions prescribed to the surrounding isodose every other day. Acute grade 3+ toxicity was 13% and all patients achieved a margin-negative resection. Median tumor-necrosis was 60% and only one patient developed local recurrence (median follow up 9 months). Planned vacuum-assisted wound closure was necessary in 30% with no other wound complications observed. Moreover, the Polish group reported a subgroup analysis of their hypofractionation study focusing on 32 patients with myxoid liposarcoma (a histological subtype with known increased radiation sensitivity) and observed 5-year LRFS and OS rates of 90% and 68% [118]. Based on these results and the findings of early radiation induced tissue changes in the histological specimens despite the short interval to surgery, they conducted a phase II trials specifically investigating short-course RT in myxoid liposarcomas followed by delayed surgery [119]. They recently published their first results, indicating low acute and late toxicity and excellent local control (100% after a median f/u of 27 months). Similar concepts are currently investigated by several groups using slightly different fractionation regimens (5–15 fractions) and single-doses (2.85–7 Gy), which are biologically equivalent to roughly 50 Gy in conventional fractionation (NCT NCT03819985, NCT02701153, NCT04425967, NCT02634710).

In non-extremity STS, local recurrence rates are still unacceptably high in many situations, even using combined approaches. Therefore, the second direction of research is aiming at increased efficacy for preoperative or definitive RT in highly-advanced STS either by dose-escalation using high-precision RT techniques like SBRT and particle therapy or by combination of RT with locally or systemically applied novel agents. SBRT has been historically reserved for small volumes present in oligometastatic disease but is currently being investigated also for primary tumors. For example, the University of Wisconsin is currently performing a phase II study using SBRT to 60 Gy in 3–8 fractions inclusive of suitable locally advanced and inoperable primary STS (NCT03972930). Particle beams are also investigated for further dose escalation because of their unique dosimetric advantages compared to photons. Yoon et al. [120] published first experiences, combining preoperative intensity modulated photon or proton therapy (median dose 50 Gy) with surgery and IORT (median dose 11 Gy) in 28 patients with retroperitoneal sarcomas. They observed a very promising 3-year LRFS rate of 90% in primary cases with low rates of radiation-related complications (14%). Recently, Delaney et al. [121] reported a phase I trial using preoperative intensity-modulated protons to deliver increased doses up to 63 Gy in 28 fractions to the region at highest risk for margin-positivity during surgery and found no dose-limiting acute toxicity. Several other groups are currently conducting phase II trials to evaluate the possible benefits of protons or carbon ions. For example, the University of Heidelberg launched a phase II trial comparing neoadjuvant particle irradiation with 39 GyE in 13 fractions in retroperitoneal sarcoma using either protons or carbon ions (NCT04219202). A US multicenter phase I/II trial also investigates neoadjuvant irradiation with integrated boost to the high-risk area in retroperitoneal sarcomas using either intensity-modulated photons or protons (NCT01659203).

Combinations with chemotherapy have been evaluated already in the past, either for preoperative or definitive treatment of high-risk STS. Probably the most recognized regimen was the combination of MAID chemotherapy with interdigitated radiation therapy introduced by the MGH group in the late 90s. Forty-eight high-risk STS patients were treated with three neoadjuvant cycles of MAID with 2 blocks of radiation therapy (each 22 Gy in conventional fractionation) in between prior to surgery and adjuvant chemotherapy [122]. Initial results were very promising, with increased local and distant control as well as survival compared to historical controls with enhanced but acceptable toxicity. However, the subsequent multicenter RTOG 9514 trial enrolling 66 patients reported high rates of toxicities including 5% deaths and 83% grade 4 side effects although achieving promising local control rates (5y-LRFS 90%) in this unfavourable patient group [123]. Small series evaluated the combination of radiation with ifosfamide, doxorubicin, or temozolomide (see definitive radiation therapy section) with promising results in resectable and unresectable STS, although at the cost of sometimes considerable and/or dose limiting toxicities, which prevented the combination regimens from becoming a widely accepted standard therapy. With new systemic possibilities like targeted agents or checkpoint inhibitors showing favourable or at least different toxicity profiles when used as a sole treatment, as well as anticipated reductions of combined toxicities by the use of modern RT techniques, combination approaches have gained new attraction. Encouraging preliminary data regarding the efficacy of combinations with targeted agents like Bevacizumab, Sorafenib, Sunitinib, and Pazopanib have been published, although sometimes accompanied by unexpectedly high toxicities [124]. At present, a variety of phase I/II trials are further investigating such combination regimens in different settings. Those include (but are not limited to) tyrosinkinase or multikinase inhibitors like sunitinib (NCT01498835) or cabozantinib (NCT04220229), PARP-inhibitors like Olaparib (NCT02787642), MDM2-inhibitors (NCT03217266), or checkpoint-inhibitors like pembrolizumab (NCT03338959) or atezolizumab (NCT03474094) with mature results pending.

Recently, intratumoral injections of radio-enhancing substances have also gained attention because of their likely minimized systemic side effects. While combinations of RT with intratumoral injections of oncolytic virus particles like T-VEC (NCT02453191) or autologeous dendritic cells (NCT01347034) are currently evaluated in phase I/II trials with pending results, encouraging evidence for intratumoral injection of nanoparticles like NBTX3 has been recently published. Radiobiological effects like ionization leading to DNA damage and cell killing are increased in tissues including large proportions of substances with high atomic number (Z). Thus, injection and selective accumulation of such substances (like hafnium oxide, *Z* = 72) in target tissues with subsequent exposure to radiation should augment cell damage to the tumor without adding toxicity to adjacent normal tissues and without involving biological pathways leading to major systemic toxicity [125]. Based on promising phase I data, Bonvalot et al. [125] conducted a phase II-III trial randomizing 180 patients to standard preoperative RT (50 Gy) with or without NBTX3 hafnium oxide nanoparticles injected into the primary tumor site once prior to start of RT. They observed a significant improvement in pCR rates (16% vs. 8%, primary endpoint) and an impressive increase in R0 resection rates (77% vs. 64%, *p* = 0.042) with no significant difference in severe side effects and concluded that nanoparticles may represent a new treatment option, although long-term results still have to be analyzed.

Finally, increasing evidence exists for a different biological behaviour and outcome of certain subtypes of STS building a rationale for specific treatment approaches, also including tailored radiation therapy. For example, myxoid liposarcomas (MLS) show distinctly improved responses to RT compared to most other subtypes. Le Grange et al. [11] described a mean diameter reduction of 21% and a mean volume reduction of 64% after preoperative RT with 50 Gy compared to 14% and 33% for all other analyzed subtypes. All MLS lesions in this series were amenable to microscopic complete resection after preoperative RT with a local control rate of 100%. Similarly, Choudry et al. [126] observed a median necrosis rate of 95% with 12% pCR after neoadjuvant irradiation with 50 Gy. Kosela-Paterczyk et al. [118] found a 5-year local control rate of 90% for MLS treated with a hypofractionated RT regime (5 × 5 Gy) and Gronchi et al. [127] recently reported partial responses in 36% according to RECIST and 86% according to Choi criteria in a phase I trial combining RT with Trabectidin. The recently published STRASS trial on neoadjuvant RT in retroperitoneal STS similarly showed clearly different outcomes according to histological subtype [30]. While the addition of RT to surgery resulted in clearly reduced rates of true local recurrences after gross total resection in liposarcomas compared to surgery alone, no benefit was observed for leiomyosarcomas [30]. Those examples show that certain subtypes of STS may be adequately treated with less aggressive or even without radiation therapy, while others need further treatment intensification, which highlights the necessity of histology-stratified trials in the future. Given the rarity of STS per se (and even more of different histological subgroups), this will require close collaborations within large-scale multicenter trials.

## 10. Conclusions/Summary

Radiation therapy plays an important role in the multimodality treatment of adult STS. In resectable, non-metastatic lesions of extremity and trunk, it is usually applied perioperatively to increase local control based randomized trials. The magnitude of its benefit depends on individual risk factors like (anticipated) resection margin, grade, size, and localization of the primary lesion. Preoperative and postoperative RT seem to result in similar oncological outcomes but implicate distinctly different toxicity profiles. While preoperative RT typically results in increased wound complication rates, late side effects impacting functional outcome are clearly decreased compared to the postoperative approach based on the smaller target volumes and lower doses needed. In retroperitoneal STS, preoperative radiation is clearly favourable compared to its postoperative use due to improved target coverage and reduced side effects. Therefore, current international guidelines discourage postoperative RT except in highly selected cases. Recent evidence suggests a reduction of local recurrences after gross complete resection with the addition of radiation to surgery, at least in liposarcomas, while leiomyosarcomas do not seem to profit and its role in other histological subgroups remains unclear. In head and neck cancer STS, postoperative radiation has been used in the majority of patients, although no data clearly discouraging preoperative RT has been published so far. Modern radiation techniques like IMRT or IGRT allow more precise radiation treatments with smaller safety margins, leading to an improved therapeutic ratio and should be used preferably. Alternative boosting techniques like IORT or brachytherapy allow further tailoring with regard to either increased efficacy or improved functional outcome. Particle beams seem advantageous in certain situations due to their unique dose profile, although mature date from large series have not been published so far. Primary, non-metastatic lesions, which are not amenable to surgery either for technical or functional reasons may be adequately treated by radiation alone. Although outcomes are clearly inferior to combined radiation and surgery, a substantial proportion of patients achieved long-term disease control. Preliminary experiences evaluating concurrent application of chemotherapy and/or hyperthermia point in the direction of improved efficacy at the cost of increased toxicity. In metastatic patients with low disease burden (oligometastatic disease), who are still amenable to curative intent treatment approaches, high-precision RT techniques like SBRT offer an efficient alternative to surgical approaches. Although no randomized trials specifically addressing its role in STS patients have been conducted, SBRT seems to result in similar local control rates and overall outcomes in patients with limited pulmonary metastases compared to surgery. Moreover, SBRT can achieve durable local control with low toxicity in patients with limited bony lesions especially in the spine where complete resections are usually not possible without major functional deficits. Similar results, although based on scarce data, have been achieved in other sites. Palliative RT can be used for either locally-advanced primary tumors or metastatic disease-causing devastating symptoms like pain or local compression. Although palliative RT is used in a substantial proportion of STS patients, little data on its efficacy specifically addressing STS patients is available. Dose and fractionation are usually based on the general rules for palliative RT regardless of histology, preferring short courses of RT with large fraction sizes and low total doses. However, recent data suggests possible benefits for moderately hypofractionated regimens with slightly increased total doses resulting in durable symptom control. Local recurrences remain a general challenge, especially in non-extremity sites were amputation is not an option. Reirradiation of local recurrences can be considered with different techniques, but the possible local control benefit has to be carefully weighed against increased late toxicities. Brachytherapy or particle therapy might be beneficial compared to photon EBRT approaches with regard to their limited irradiated volumes. Future directions of research include the use of more convenient, shorter fractionation regimes during the perioperative approach especially in radiosensitive histologies and the introduction of particle beams like protons or carbon ions for the preoperative or definitive treatment aiming at increased local control and reduced toxicity based on the promising results from chondrosarcoma or chordoma series. Enhancement of radiation efficacy is further evaluated in a variety of phase I/II trials combining RT with targeted agents or checkpoint inhibitors. Moreover, novel intratumoral applied radiation-enhancers like nanoparticles have gained attraction due to their unique mode of action based on the encouraging results from a recent phase II/III trial. Increasing evidence further suggests the necessity of tailoring radiation therapy according to histological subtype instead of the current one size fits all approach which highlights the need for close multicenter collaborations in future trials based on the rarity of the disease.

## Figures and Tables

**Table 1:** Major series of patients with pulmonary metastases of sarcoma treated by SBRT.

Author	Year	Med. f/u	N (les.)	Fractionation	2y-LC	2y-OS	gr. 3+ tox.
Dhakal [81]	2012	11	15 (74)	50 Gy/5 Fx	82% ^3^	25 mo ^6^	None
Mehta [82]	2013	20	16 (25)	54 Gy/3–4 Fx	94% ^3^	72% ^3^	None
Frakulli [83]	2015	17	24 (68)	30–60 Gy/3–8 Fx	86%	66%	n.r.
Navarria [84]	2015	21	28 (51)	48 Gy/4 Fx ^1^	96% ^4^	61% ^4^	none
Baumann [85]	2016	16	30 (39)	50 Gy/4–5 Fx	86%	43%	none
Lindsay [86]	2017	14	44 (117)	50 Gy/10 Fx	95% ^5^	82%	2%
Soyfer [87]	2017	95	22 (34)	60 Gy/3 Fx ^2^	100% ^4^	50% ^4^	n.r.
Baumann [88]	2020	16	44 (56)	50 Gy/4–5 Fx	90%	46%	none

Med. f/u: median follow-up in months, n (les.): number of patients (lesions), fractionation: total dose and number of fractions, in series that included different fractionation schedules, the most common is reported, 2y-LC: 2-year local control rate (if not otherwise specified), 2y-OS: 2-year overall survival rate (if not otherwise specified), gr. 3+ tox.: grade 3 or higher toxicity according to the grading system used in the study, n.r.: not reported, Gy: Gray, Fx: number of fractions, ^1^ fractionation was adapted to lesion size and localisation (used schedules included 30 Gy/1 Fx, 60 Gy/20 Fx, 60 Gy 8/Fx and 48 Gy/4 Fx, all BED > 100 with alpha/beta 10, ^2^ dose calculation without inhomogeneity calculation, ^3^ 3-year rate, ^4^ 5-year rate, ^5^ crude rate, ^6^ median overall survival.
